# OpenNahele: the open Hawaiian forest plot database

**DOI:** 10.3897/BDJ.6.e28406

**Published:** 2018-09-27

**Authors:** Dylan Craven, Tiffany M Knight, Kasey E Barton, Lalasia Bialic-Murphy, Susan Cordell, Christian P Giardina, Thomas W Gillespie, Rebecca Ostertag, Lawren Sack, Jonathan M Chase

**Affiliations:** 1 Department of Community Ecology, Helmholtz Centre for Environmental Research - UFZ, Halle (Saale), Germany Department of Community Ecology, Helmholtz Centre for Environmental Research - UFZ Halle (Saale) Germany; 2 German Centre for Integrative Biodiversity Research (iDiv) Halle-Jena-Leipzig, Leipzig, Germany German Centre for Integrative Biodiversity Research (iDiv) Halle-Jena-Leipzig Leipzig Germany; 3 University of Göttingen, Göttingen, Germany University of Göttingen Göttingen Germany; 4 Institute of Biology, Martin Luther University Halle-Wittenberg, Halle (Saale), Germany Institute of Biology, Martin Luther University Halle-Wittenberg Halle (Saale) Germany; 5 Department of Botany, University of Hawai'i at Mānoa, Honolulu, United States of America Department of Botany, University of Hawai'i at Mānoa Honolulu United States of America; 6 Department of Ecology and Evolutionary Biology, University of Tennessee Knoxville, Knoxville, United States of America Department of Ecology and Evolutionary Biology, University of Tennessee Knoxville Knoxville United States of America; 7 Institute of Pacific Islands Forestry, USDA Forest Service, Hilo, United States of America Institute of Pacific Islands Forestry, USDA Forest Service Hilo United States of America; 8 University of California Los Angeles, Los Angeles, United States of America University of California Los Angeles Los Angeles United States of America; 9 Department of Biology, University of Hawai‘i at Hilo, Hilo, United States of America Department of Biology, University of Hawai‘i at Hilo Hilo United States of America; 10 Department of Ecology and Evolutionary Biology, University of California Los Angeles, Los Angeles, United States of America Department of Ecology and Evolutionary Biology, University of California Los Angeles Los Angeles United States of America; 11 Department of Computer Science, Martin Luther University, Halle, Germany Department of Computer Science, Martin Luther University Halle Germany

## Abstract

**Background:**

This data paper provides a description of OpenNahele, the open Hawaiian forest plot database. OpenNahele includes 530 forest plots across the Hawaiian archipelago containing 43,590 individuals of 185 native and alien tree, shrub and tree fern species across six islands. We include estimates of maximum plant size (D95_0.1_ and D_max3_) for 58 woody plant species, a key functional trait associated with dispersal distance and competition for light. OpenNahele can serve as a platform to test key ecological, evolutionary and conservation questions in a hotspot archipelago.

**New information:**

OpenNahele is the first database that compiles data from a large number of forest plots across the Hawaiian archipelago to allow broad and high resolution studies of biodiversity patterns.

**Keywords**: Hawaii, forests, islands, biodiversity, community ecology, evolutionary ecology

## Introduction

Oceanic islands are hotspots of species endemism and biodiversity that contain an estimated 17% of the world’s plant diversity on just 5% of its area (Kier et al. 2009, Tershy et al. 2015). Biodiversity on islands is increasingly threatened by alien species (Sax and Gaines 2008, Dawson et al. 2017), which also may affect the ability of island ecosystems to provide vital ecosystem services. Amongst island systems, Hawai’i is amongst the most intensively studied and has been used as a model system to test fundamental ecological and evolutionary questions (e.g. Vitousek et al. 1987, Gillespie 2004, Price 2004, Gruner 2007, Givnish et al. 2009, Rominger et al. 2016). However, our understanding of biodiversity patterns across the Hawaiian archipelago remains limited (even for well-studied taxa such as plants) because open access data are available at coarse scales (e.g. species checklists for islands) but not at the community-level scales that are relevant to many ecological and evolutionary questions.

While coarse-scale data are used in macroecological studies that examine biodiversity patterns across islands globally (e.g. Kreft et al. 2008), finer-scale data are necessary to understand patterns of community structure, i.e. which species are dominant or rare and species responses to natural and anthropogenic drivers. Community data, comprised of abundances of individual species in a discrete area, address many of the shortcomings of coarse-scale data. Unfortunately, fine-scale community data from individual studies are collected across small spatial extents with low sampling intensities (usually for logistical reasons), which has prevented the analysis of within-island biodiversity patterns across multiple islands in the Hawaiian archipelago (but see Rominger et al. 2016 for a study on arthropods). There have been recent calls for open access forest plot monitoring data (Borges et al. 2018) and, to our knowledge, this database will be the first community-level database for the Hawaiian archipelago.

## General description

### Purpose

To facilitate the analysis of biodiversity patterns within and across islands in Hawaiian forests, we present the OpenNahele database ('nahele' means forest in Hawaiian). This database compiles forest plot data from all six major islands of the Hawaiian archipelago and contains 530 plots, 185 tree, shrub and tree fern species and 43,590 individuals (Tables 1, 2). The size of each individual (diameter at 1.3 cm; DBH) is included in the database. For each species, we also include information about whether the species is native or alien to the Hawaiian archipelago and whether the species is cultivated or not. We harmonised taxonomic names, species abundance and individual size from multiple studies to facilitate the calculation of diversity metrics that are comparable. Additionally, we used individual size data to estimate maximum Maximum plant size for 58 woody plant species. Adult plant size is a key plant functional trait that is strongly related to dispersal distance (Thomson et al. 2011) and competition for light (King et al. 2006).

**Figure 1.**
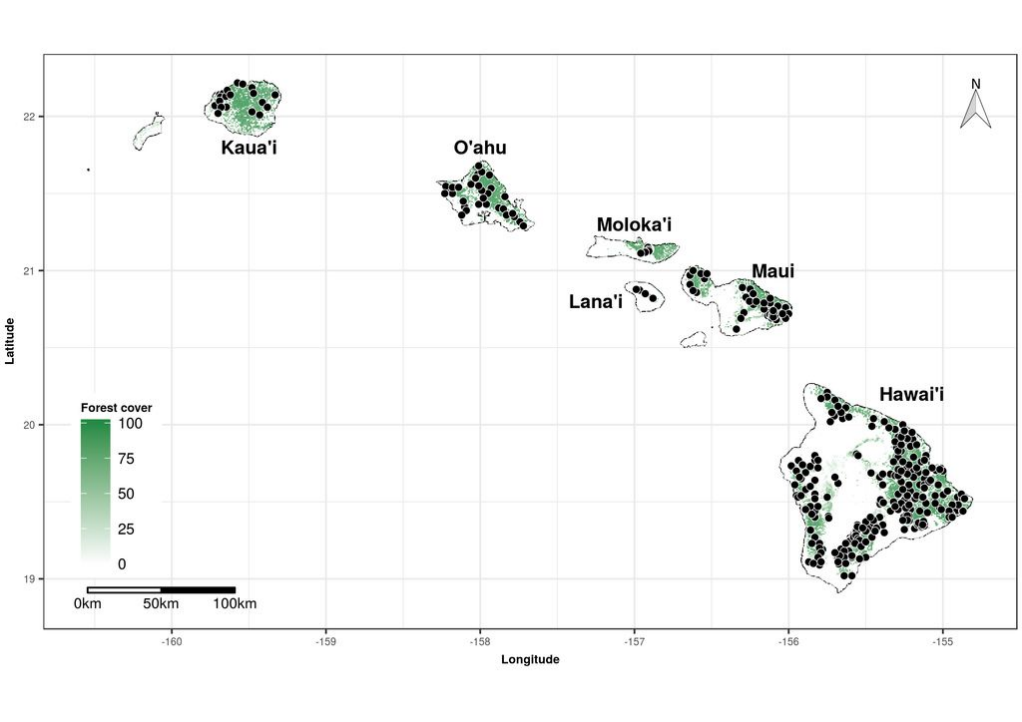
Distribution of forest plots (n = 530) across the Hawaiian archipelago. Dots represent the location of each plot in the OpenNahele database. Forest cover data from Hansen et al. (2013).

**Figure 2.**
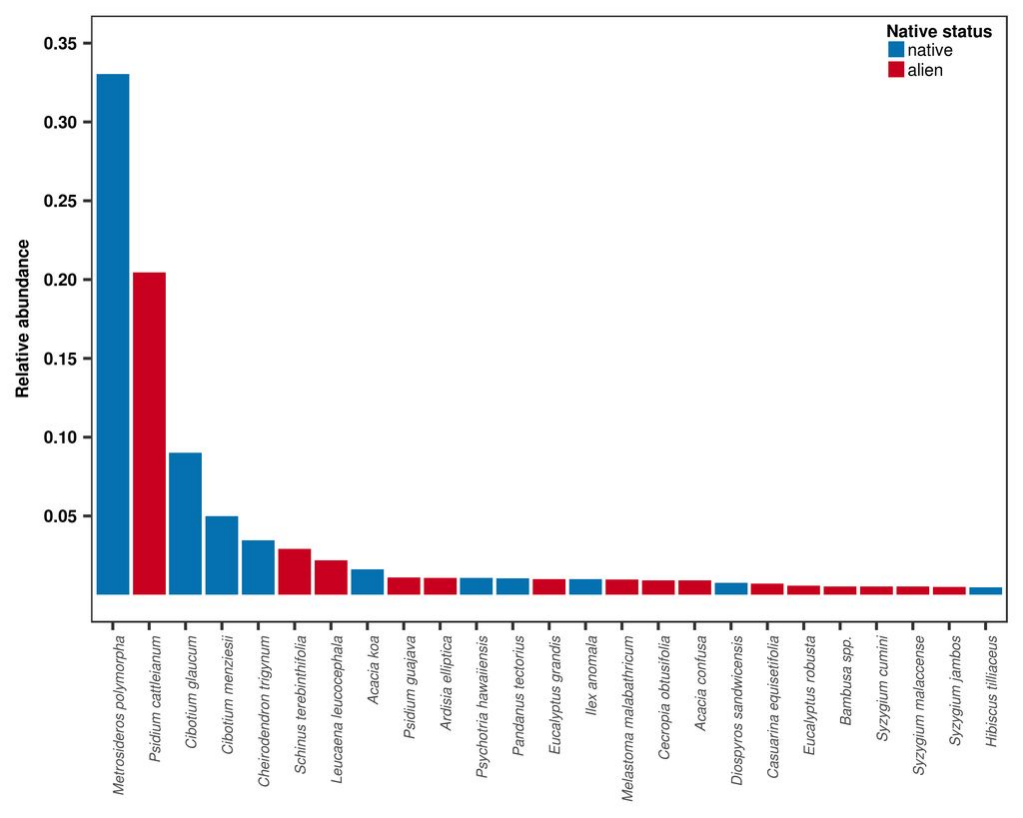
Relative abundance (proportion of individuals) of the 25 most abundant tree, shrub and tree fern species across the Hawaiian archipelago in the OpenNahele database.

The OpenNahele database can be used to examine cross-scale biodiversity patterns and drivers of and threats to biodiversity across the Hawaiian archipelago. This database provides unprecedented geographic coverage across Hawaiian forests (Fig. 1). While this database captures only a fraction of the entire Hawaiian flora, it provides a realistic snapshot of the current state of Hawaiian forests. For example, this database shows that only a few native and alien species are dominant in Hawaiian forests, such as *Metrosideros polymorpha*, a native tree in the Myrtaceae family (Fig. 2; Table 3). The database also reveals that alien invasions are widespread across Hawaiian forests, having occurred on all major islands and in 45% of plots (Table 4). While alien species represent 11.7 % of individuals in the database, some species are highly abundant in the plots where they occur. The median abundance of stems of alien species in invaded plots is 44.5%, but varies markedly across islands from 5% on Lana’i to 87% on O’ahu (Table 4). As sampling effort and plot size varies within the database, we provide relative measures of abundance that are standardised to a common area (see Sampling methods) to examine community structure and caution against using raw measures of abundance, as they may introduce bias to diversity indices that are sensitive to the number of individuals.

**Figure 3.**
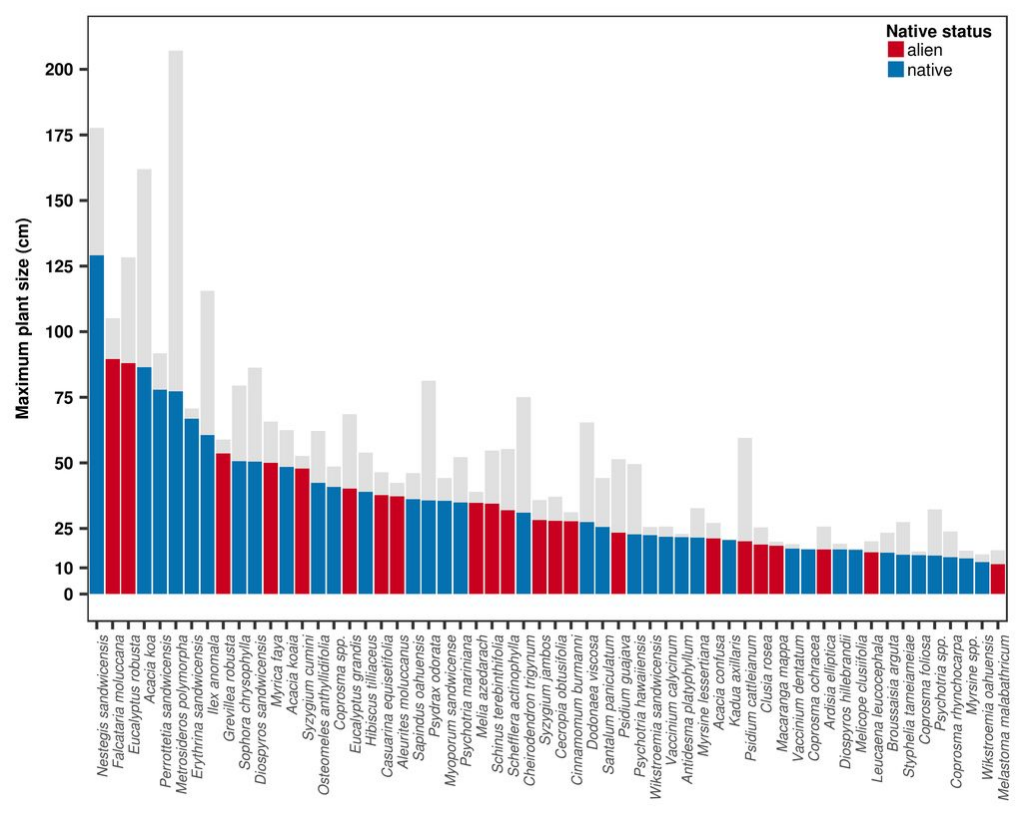
Maximum plant size of 58 plant species across the Hawaiian archipelago. Maximum plant size was estimated from individuals = 5 cm diameter using two methods: i) as D95_0.1_ (shown in colour), which is the 95th percentile of stem diameter of all diameters = 0.1 x maximum observed diameter and ii) D_max3_ (shown in light grey), which is the average diameter of the three largest individuals across the OpenNahele database.

The OpenNahele database also can be used to explore ecological differences amongst species. For example, adult plant size can be used to assess the extent to which species’ geographic ranges are related to dispersal or if they are limited by habitat availability (Fig. 3; Gaston and Fuller 2009).

The OpenNahele database will be maintained and curated as data from future censuses and new studies become available to capture temporal dynamics of populations and communities across Hawaiian forests.

## Sampling methods

### Study extent

To compile a database of plots in Hawaiian forests (Suppl. material 1), we consulted local experts with extensive experience in forestry, community ecology and botany, as well as experts with knowledge of former and ongoing research projects in Hawai’i, USA. From an initial list of publicly available sources and published studies, we examined each to determine if they met our inclusion criteria. Our inclusion criteria were that each study reports for each plot: i) geographical location, ii) species identity and iii) abundance as the number of individuals of trees, shrubs, and tree ferns. We downloaded raw data or obtained it directly from data owners, which also included individual size either as diameter at 1.3 m (diameter at breast height; DBH) or in size classes. In total, we identified 6 unique studies meeting our inclusion criteria that together comprise 43,590 individuals of 185 species within 530 plots across six islands (Table 1; Zimmerman et al. (2007), Gillespie et al. (2013), Ostertag et al. (2014), Knight & Barton (unpublished), National Park Service’s (NPS) Pacific Island Network (PACN; Ainsworth et al. 2011) and the publicly available data from the U.S. Forest Service (USFS) Forest Inventory & Analysis (FIA) program in Hawaii (US Forest Service 2016). Data were collected between 2003 and 2015.

### Sampling description

Studies in the OpenNahele database used different plot sizes and minimum size thresholds (Table 1). Plot sizes ranged from 12.97 m^2^ to 40,000 m^2^ and the median plot size across the database is 1,000 m^2^. Half of the studies used one minimum size threshold and included all individuals above that in their inventory. The other half of the studies used a nested sampling approach, whereby smaller subplots were placed within each plot to assess individuals below the size threshold of the full plot. The minimum size threshold varied across studies from 1 to 2.54 cm DBH. Currently, all studies have only conducted one inventory. Geographic coordinates of all forest plots were converted to the WGS84 coordinate system, a standard coordinate system with a spheroidal reference surface, to facilitate the retrieval of climate, topographical and geological data. Locations of plots in the USFS FIA were fuzzed up to 1.6 km of their exact locations (US Forest Service 2016), but those of other studies were not altered. Geographic coordinates were checked visually.

Most studies inventoried trees, shrubs and tree ferns. While tree ferns do not have true wood, they play an important ecological role in Hawaiian forests (Zimmerman et al. 2008). One study, Knight & Barton, did not record the presence of tree ferns (e.g. *Cibotium* spp., *Sadleria* spp.). However, only two plots in that study were located in areas where tree ferns occur and there are many other nearby plots in the database that included tree ferns. As not all studies included lianas, i.e. woody vines, we removed species classified as such by USDA Plants (USDA NRCS 2018).

Data from one study within the OpenNahele database, HIPPNET (Ostertag et al. (2014)), are curated on an ongoing basis and expanded with data from subsequent censuses; interested data users should contact HIPPNET via the CTFS-ForestGEO website.

*Abundance*

To facilitate aggregation of abundance data across studies that differ in plot size, we calculated abundance of individuals per species on a per-hectare basis:

Abundance per ha = Abundance / Area *x* 10,000

where abundance is the number of individuals per species and Area is the plot (or sub-plot) area in square metres.

Individual size was converted to centimetres if measured as DBH or classified as greater than or less than 5 cm DBH if individual size was not measured. As data sources used different minimum DBH thresholds, which may influence the number of individuals in a plot in a systematic way, i.e. plots with larger DBH thresholds will have fewer individuals than those with smaller DBH thresholds and, therefore, species diversity estimates, we removed individuals smaller than 5 cm DBH. To further account for variation in the number of individuals due to differences in plot area across the database, we recommend estimating species diversity based on rarefaction curves (e.g. Chao et al. 2014, Chase et al. 2018).

*Maximum plant size*

We estimated maximum plant size for individual species in two ways: as the 95th percentile of stem diameter of all diameters > 0.1 x maximum observed diameter (D95_0.1_) and as the mean diameter of the three largest individuals across the database (D_max3_; King et al. 2006). In total, we estimated both measures of maximum plant size for 58 woody plant species that had at least 20 individuals = 5 cm DBH (Suppl. material 2). We compared both measures using Spearman’s correlation coefficient and found that while strongly and positively correlated (*rho* = 0.89, *p*-value< 0.001), D_max3_ was on average 49% larger than D95_0.1_. As D95_0.1_ is not sensitive to sample size (King et al. 2006), we recommend using this measure over D_max3_, particularly for studies using it as a functional trait in combination with species abundance data.

### Quality control

Taxonomic names were resolved and harmonised with The Plant List v. 1.1 (The Plant List 2013) using the ‘Taxonstand’ package (Cayuela et al. 2017). Family names and orders were also retrieved and used to identify angiosperms and monocots following the Angiosperm Phylogeny Group III (Angiosperm Phylogeny Group 2009). Native status was obtained by consulting the electronic Flora of Hawaii (Wagner et al. 2005). Individuals not identified to the species level were classified as ‘uncertain’, unless the genus was endemic to Hawai’i. As not all introduced species have been naturalised, i.e. they are cultivated in forestry plantations or as ornamentals but have yet to establish self-sustaining populations, we also obtained the cultivation status for introduced species using the Pacific Island Ecosystems at Risk database (US Forest Service 2017). For each individual, accepted species name, family, angiosperm and monocot classification, native status and cultivated status are provided (Suppl. material 1).

## Geographic coverage

### Description

The 530 plots in the OpenNahele database are located on all six major islands of the Hawaiian archipelago (Fig. 1). Forested areas are well covered by plots on most islands and include a wide range of habitat types, from tropical dry forests to subalpine shrublands. However, not all islands were sampled with the same intensity (Table 2); 380 plots are located on the largest island, Hawai’i, and the other, smaller islands have between 5 and 59 plots. One potential limitation with this database is that most studies, with the exception of those collected by the USFS FIA, did not locate plots randomly, a fact which may introduce a bias towards forests with relatively low amounts of alien species.

### Coordinates

18.90986 and 22.23583 Latitude; -154.8058 and -160.5458 Longitude.

## Taxonomic coverage

### Description

In total, the OpenNahele database contains 185 tree, shrub and tree fern species, of which 61% and 39% are native and alien, respectively, and which represent 16% of the 1,155 woody species that occur across the Hawaiian archipelago (Wagner et al. 2005). The database captures a relatively small proportion of woody species in Hawai’i, possibly because many species may not reach the minimum size limit (5 cm DBH) and that plots were only located in forests and not in ecosystems where woody species occur but are small in stature or are not dominant, e.g. shrublands and grasslands. However, the database has a similar proportion of alien woody species (39%) as found across the entire Hawaiian archipelago (41%; Wagner et al. 2005).

*Dominant species*

*Metrosideros polymorpha* is hyperdominant in Hawaiian forests and represents 33% of all individuals greater than 5 cm DBH (Fig. 2; Table 3). Two species of native tree ferns, *Cibotium glaucum* and *C. menziesii*, are amongst the five most abundant species in the database. Alien species have heavily invaded Hawaiian forests; fifteen of the 25 most abundant species in the database are alien (Table 3).

## Usage rights

### Use license

Creative Commons Public Domain Waiver (CC-Zero)

## Data resources

### Data package title

OpenNahele: the open Hawaiian forest plot database

### Resource link

https://doi.org/10.5061/dryad.1kk02qr

### Number of data sets

2

### Data set 1.

#### Data set name

OpenNahele forest plot data

#### Number of columns

18

#### Description

Diameter at breast height (or occurrence) of individual trees, shrubs and tree ferns across 530 plots across the Hawaiian archipelago and includes native status and cultivated status of the 185 species. Available as (Suppl. material 1)

**Data set 1.** 

Column label	Column description
Island	Island name
PlotID	Unique numeric identifier for each plot
Study	Brief name of study
Plot_area	Plot area in m^2^
Longitude	Longitude of plot in decimal degrees; WGS84 coordinate system
Latitude	Latitude of plot in decimal degrees; WGS84 coordinate system
Year	Year in which plot data was collected
Census	Numeric identifier for each census
Tree_ID	Unique numeric identifier for each individual
Scientific_name	Genus and species of each individual following TPL v. 1.1
Family	Family of each individual following TPL v. 1.1
Angiosperm	Binary variable (1 = yes, 0 = no) indicating whether an individual is classified as an angiosperm following APG III
Monocot	Binary variable (1 = yes, 0 = no) indicating whether an individual is classified as a monocot following APG III
Native_Status	Categorical variable (‘native’, ‘alien’, ‘uncertain’) indicating alien status of each individual following Wagner et al. (2005)
Cultivated_Status	Binary variable (1 = yes, 0 = no, NA = not applicable) indicating if species is cultivated following PIER
Abundance	Number of individuals (all = 1)
Abundance_ha	Abundance of each individual on a per hectare basis
DBH_cm	Diameter at 1.3 m (DBH) for each individual; NA indicates that size was not measured, but was classified by size class

### Data set 2.

#### Data set name

OpenNahele Maximum Plant Size

#### Number of columns

6

#### Description

Maximum plant size of 58 tree, shrub and tree fern species that occur in 530 forest plots across the Hawaiian archipelago. Maximum plant size was estimated as D95_0.1_and D_max3_ following King et al. (2006). Available as Suppl. material 2.

**Data set 2.** 

Column label	Column description
Scientific_name	Genus and epithet of each individual following The Plant List v. 1.1 (2013)
Family	Family of each individual following The Plant List v. 1.1 (2013)
Native_Status	Categorical variable (‘native’, ‘alien’, ‘uncertain’) indicating alien status of each individual following Wagner et al. (2005)
N	Number of individuals used to estimate maximum plant size
D95	Maximum plant size, estimated as D950.1 (King et al. 2006)
Dmax_3	Maximum plant size, estimated as Dmax3 (King et al. 2006)

## Supplementary Material

Supplementary material 1 OpenNahele forest plot data Data type: Size and occurrences of individual trees, shrubs and tree ferns
Brief description: Diameter at breast height (or occurrence) of individual trees, shrubs and tree ferns across 530 plots across the Hawaiian archipelago and includes native status and cultivated status of the 185 species.
File: oo_217337.csv Dylan Craven, Tiffany M. Knight, Kasey E. Barton, Lalasia Bialic-Murphy, Susan Cordell, Christian P. Giardina, Thomas W. Gillespie, Rebecca Ostertag, Lawren Sack, Jonathan Chase

Supplementary material 2 OpenNahele Maximum Plant Size Data type: Maximum size
Brief description: Maximum plant size of 58 tree, shrub and tree fern species that occur in 530 forest plots across the Hawaiian archipelago. Maximum plant size was estimated as D950.1 and Dmax3 following King et al. (2006).
File: oo_217338.csv Dylan Craven, Tiffany M. Knight, Kasey E. Barton, Lalasia Bialic-Murphy, Susan Cordell, Christian P. Giardina, Thomas W. Gillespie, Rebecca Ostertag, Lawren Sack, Jonathan Chase

## Figures and Tables

**Table 1.** Basic description of forest plot data for each data source in OpenNahele.

**Data Source**	**Islands (#)**	**Plots (#)**	**Plot size (m^2^)**	**Min. DBH (cm)**	**Individuals (#)**	**Species (#)**
Gillespie et al. 2013	6	15	1000	2.5	1836	56
Ostertag et al. 2014	1	2	40000	1	14365	30
Knight & Barton	5	35	1000	1	2631	74
NPS PACN	3	150	1000	1/10	13055	41
FIA Hawaii	5	234	13-672.5	2.54/12.7	6898	102
Zimmerman et al. 2007	1	94	100-1017.9	2/30	4805	28

**Table 2.** Sampled area, number of plots and number of individuals and species per island in OpenNahele.

**Island**	**Sampled area (m^2^)**	**Plots (#)**	**Individuals (#)**	**Species(#)**
Hawai’i Island	359380	380	31509	114
Kaua’i Island	15388	22	1425	67
Lana’i Island	3841	5	426	11
Maui Island	47945	59	3980	82
Moloka’i Island	32000	32	4985	31
O’ahu Island	19940	32	1265	59

**Table 3.** Relative abundance (proportion of individuals) of the 25 most abundant tree, shrub and tree fern species across the Hawaiian archipelago in OpenNahele.

**Species**	**Native status**	**Relative abundance**
*Metrosideros polymorpha*	native	0.33
*Psidium cattleianum*	alien	0.20
*Cibotium glaucum*	native	0.09
*Cibotium menziesii*	native	0.05
*Cheirodendron trigynum*	native	0.03
*Schinus terebinthifolia*	alien	0.03
*Leucaena leucocephala*	alien	0.02
*Acacia koa*	native	0.02
*Psidium guajava*	alien	0.01
*Ardisia elliptica*	alien	0.01
*Psychotria hawaiiensis*	native	0.01
*Pandanus tectorius*	native	0.01
*Eucalyptus grandis*	alien	0.01
*Ilex anomala*	native	0.01
*Melastoma malabathricum*	alien	0.01
*Cecropia obtusifolia*	alien	0.01
*Acacia confusa*	alien	0.01
*Diospyros sandwicensis*	native	0.01
*Casuarina equisetifolia*	alien	0.01
*Eucalyptus robusta*	alien	0.01
*Bambusa*spp.	alien	0.01
*Syzygium cumini*	alien	0.01
*Syzygium malaccense*	alien	0.01
*Syzygium jambos*	alien	0.00
*Hibiscus tilliaceus*	native	0.00

**Table 4.** Proportion of invaded plots and median relative abundance (% of individuals) of alien plant species in invaded plots per island across the Hawaiian archipelago in OpenNahele.

**Island**	**Proportion of Invaded plots**	**Median relative abundance of alien species**
Hawai’i Island	0.40	0.39
Kaua’i Island	0.82	0.76
Lana’i Island	1.00	0.19
Maui Island	0.49	0.66
Moloka’i Island	0.06	0.05
O’ahu Island	0.94	0.87
